# Deploying machine learning with messy, real world data in low- and middle-income countries: Developing a global health use case

**DOI:** 10.3389/fdata.2022.553673

**Published:** 2022-07-27

**Authors:** Amy Finnegan, David D. Potenziani, Caroline Karutu, Irene Wanyana, Nicholas Matsiko, Cyrus Elahi, Nobert Mijumbi, Richard Stanley, Wayan Vota

**Affiliations:** ^1^IntraHealth International, Chapel Hill, NC, United States; ^2^Duke Global Health Institute, Durham, NC, United States; ^3^IntraHealth International, Mbale, Uganda; ^4^Makerere University School of Public Health, Kampala, Uganda

**Keywords:** machine learning, Uganda, health informatics, low- and lower-middle-income countries, data analytics

## Abstract

The rapid emergence of machine learning in the form of large-scale computational statistics and accumulation of data offers global health implementing partners an opportunity to adopt, adapt, and apply these techniques and technologies to low- and middle-income country (LMIC) contexts where we work. These benefits reside just out of the reach of many implementing partners because they lack the experience and specific skills to use them. Yet the growth of available analytical systems and exponential growth of data require the global digital health community to become conversant in this technology to continue to make contributions to help fulfill our missions. In this community case study, we describe the approach we took at IntraHealth International to inform the use case for machine learning in global health and development. We found that the data needed to take advantage of machine learning were plentiful and that an international, interdisciplinary team can be formed to collect, clean, and analyze the data at hand using cloud-based (e.g., Dropbox, Google Drive) and open source tools (e.g., R). We organized our work as a “sprint” lasting roughly 10 weeks in length so that we could rapidly prototype these approaches in order to achieve institutional buy in. Our initial sprint resulted in two requests in subsequent workplans for analytics using the data we compiled and directly impacted program implementation.

## Introduction

Machine learning and artificial intelligence pose the ability for global health practitioners to glean new insights from data they are already collecting as part of implementing their programs. To date, little practice-based research has been documented on how to incorporate machine learning into international development programs.

In this community case study, we present the results of a pilot machine learning effort conducted by IntraHealth International. IntraHealth is a global health NGO based in Chapel Hill, North Carolina with active programs in over 20 countries around the world that focus on ready, connected, and safe frontline health workers as critical to ending the HIV epidemic and ensuring women everywhere have access to contraception that can help them achieve their reproductive goals. The aim of this community case study is to describe the results of a machine learning pilot at IntraHealth International to determine the amount of time, effort, and team composition that is necessary to incorporate machine learning into our field programs.

## Background and rationale

The growth of data worldwide across various domains, including health, offers new opportunities for exploration using “big data” methods such as machine learning. The digitization of health data in terms of services delivered; facility characteristics; health worker status, training, and certification; logistics information; laboratory results and medical records has increased the data available for analysis tremendously. Outside of the health domain there is a widening pool of digital resources that offer access to relatable data on weather, transportation networks, topography, population demographics and economic activity. Together, these resources offer a new opportunity to harness such heterogeneous sources of data for analysis using machine learning technologies. The challenges are where and how to begin, and how to relate these approaches to the ground-level needs of those running health care programs.

The rise of information systems supporting health care in LMICs has vexed health officials with a proliferation of systems leaving some “data rich, but information poor” (Peters and Waterman, 1982). Largely unexplored and unexploited is the promise for using such big data for advanced analytics. While dozens of LMIC ministries of health have a plethora of systems generating data, the data use value derived is usually linear in calculating arithmetical indicators for presentation on dashboards and periodic reports. Such approaches mirror in form and format the use of manually completed paper records to create periodic reports for leadership where clerks counted data points and calculated the results. As noted by Burke (2013), these reporting practices formed the base of analysis in health care systems but also have the lowest impact on decision-making for health care. Our hope was to reach a level somewhere between forecasting and predictive modeling that exponentially adds value to mere clerical data collection, thus improving knowledge discovery from such data sources.

## Methods

IntraHealth staff sought to enter the domain of advanced analytics through machine learning in a step-by-step fashion. We organized an international, multidisciplinary team to build a “data lake” that could be filled with data and later queried. We embarked on this “journey without a destination” in order to understand the time, effort, and resources needed to stand up a team and quickly build a pool of data for further analysis.

### Identifying a program partner

We arranged for access to data from a current IntraHealth-led project, the USAID-funded Regional Health Integration to Enhance Services in Eastern Uganda (USAID RHITES-E) Activity (IntraHealth International., 2017), part of a larger USAID-funded effort to support the Government of Uganda to expand accessibility and use of high-quality health services. USAID RHITES-E's main focus is building the capacity of districts in eastern Uganda to improve facility service delivery and make data-driven decisions. The USAID RHITES-E activity region includes 30 districts, two of which are in the northern Karamoja sub-region. USAID RHITES-E covers a heterogenous population that ranges from extremely rural pastoral areas to larger industrial city centers (Uganda Bureau of Statistics, 2014). In the USAID RHITES-E region, services lag behind other areas of Uganda in the midst of high poverty and food insecurity especially in the Karamoja sub-region. IntraHealth's Chief of Party for USAID RHITES-E allowed us to pursue this exploratory analysis without expecting any specific deliverable and IntraHealth funded the effort through indirect resources.

### Organizing a team

We built an interdisciplinary team based in the US and Uganda (see Table 1). The US-based team consisted of a project manager, senior data scientist, global health graduate student, and analytics expert. The Uganda-based team consisted of an informatics graduate student, monitoring and evaluation professional on the RHITES-E activity, and information and communications (ICT) technologist.

**Table 1:** Team composition, expertise, and roles.

**Team member**	**Expertise**	**Role**
Project manager (US-based)	Informatics professional with 20+ years of experience designing and deploying information management systems	Overall leadership in keeping the project on track to meet weekly and project goals; provide vision and long-term strategy development and incorporation into IntraHealth's work
Senior data scientist (US-based)	PhD-level data scientist with 10 years of experience working in global population health and demography and 3 years of machine learning experience	Provide technical leadership on data sources to include, data cleaning, machine learning methods to employ, primary development and maintenance of the code base, and presentation of results
Global health graduate student (US-based)	Second year master's student in global health at a US university who had been involved in prior machine learning projects in global health settings	Conduct literature review, co-produce an analytical memo to review machine learning strategies, and provide suggestions to the team for analytical approaches
Analytics expert (US-based)	Analyst and product manager for open health information systems with 20+ years of experience designing and developing data collection and management systems	Provide insight on how to access data systems and provide data for use in the analysis, interpretation of results
Informatics graduate student (Uganda-based)	Second year master's student in health informatics who had completed machine learning coursework at a Uganda-based university	Data aggregation, cleaning, analysis, and interpretation
Monitoring and evaluation professional (Uganda-based)	Monitoring and evaluation professional with 10 years of experience working in public health in Uganda	Provide expertise in family health in Uganda and data management and analysis support
Information and communications technologist (Uganda-based)	Informatics specialist with 8 years of experience designing, developing, and deploying informatics systems in LMIC settings	Provide insight on how to access native systems and support on understanding reasons for missing data

No team member was full time for the project.

We employed graduate students because they could bring energy and a lack of preconceived notions of what the endeavor should be. They also did much of the unglamorous work to prepare data by assessing its quality and addressing related issues. The data scientist and analytics expert offered a high level of knowledge, skills, and abilities to guide our efforts on a technical level. The ICT staff provided both knowledge of Uganda's national health information systems as well as methods to access the data effectively and responsibly. Finally, the project manager provided coordination and the occasional naive question to prompt reflection.

### Planning the work

**Figure 1:**
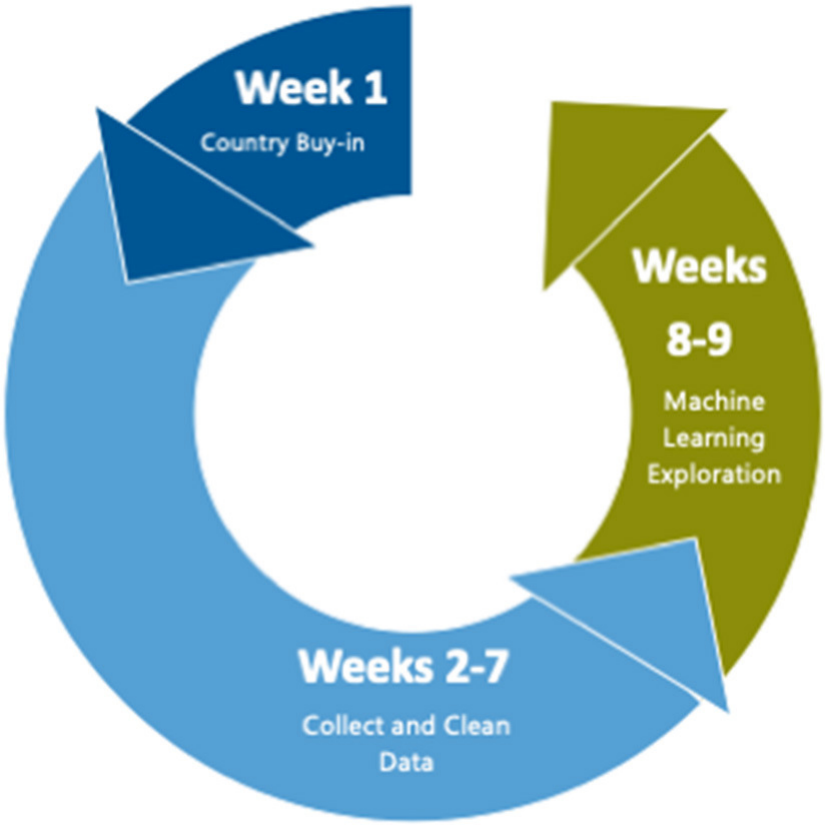
Time allocation during pilot. The first week was spent obtaining buy–in and assembling the team, weeks 2–7 were spent collecting, cleaning data, and linking data, and weeks 8–9 were spent developing and running the machine learning models.

We organized our work into a “sprint” lasting no more than 10 weeks and with less than full-time funding for the team members. The first week was spent obtaining buy-in and assembling the team, weeks 2–7 were spent collecting, cleaning, and linking data, and weeks 8–9 were spent developing the machine learning models (see Figure 1).

Our motivation for such a limited effort, in time and resources, was that for this exploratory analysis the team faced a classic “Catch-22” where the proof of utility required a leap of faith in terms of allocation of resources, both financial and intellectual. Moreover, such an effort had to be designed as a multi-disciplinary collaboration in which a variety of participants could offer their expertise and effort.

### Software

We anticipated that our data lake would grow exponentially over the course of our 10-week project. We first needed to understand where to store and how to access the data. After considering cloud resources such as Amazon Web Services (AWS), the team settled on a simpler, more accessible solution—Dropbox. Dropbox offered the type of file and directory resources needed for versioning the data, a common access point behind an authentication barrier, and enough storage to accommodate the data we intended to accumulate. We also relied on Google Drive and GitHub, an online web platform where users can submit their code to repositories with version control capabilities. These cloud-based resources provided our international team with the ability to have the most recent version of the data and code at hand for analysis.

Team members were experts in various software for data analysis including R (R Core Team, 2016), Stata, and Excel. The technical lead was fluent in R, which offers several robust packages for machine learning and the ability to read in data from multiple sources including Excel sheets, csv files, geospatial data, Stata datasets, Google Sheets, and application programming interfaces (APIs), among others. Therefore, R was used for performing the analysis and much of the data cleaning. R scripts were stored in a private GitHub repository that the team accessed.

### Collecting and cleaning the data

Almost immediately, we were able to begin filling our data lake with USAID RHITES-E activity data from a baseline facility survey conducted in 2018 and existing de-identified population data downloaded from publicly available resources. To the USAID RHITES-E activity baseline facility survey, the team added Ugandan census data from 2014 with granularity down to the parish level, the lowest political unit in Uganda; periodic raw data from the Demographic and Health Surveys (DHS) conducted from 1988 to 2016 and was available at the samplin cluster level; facility-level data from DHIS2 (Dehnavieh et al., 2019), the District Health Information System that stores data on health sector service delivery; iHRIS (IntraHealth International., 2020), a human resources information system that tracks employees at health facilities; and even meteorological data on rainfall by location obtained through the DHS spatial data repository at the sampling cluster level (The DHS Program, 2020) (see Table 2).

**Table 2:** Data sources included in data lake.

**Data type**	**Unit of analysis**	**Data source**
Program data	—Health facility	—USAID RHITES-E baseline facility assessment survey
Population representative data	—Sampling cluster —Parish	—Demographic and Health Survey (DHS) —Census
Service delivery data	—Health facility	—District Health Information System 2 (DHIS2)
Staffing data	—Health facility —Health facility type	—iHRIS (human resources information system) —Establishments data (facility staffing quotas)
Geographic data	—5 × 5 km estimates —1 × 1 km estimates	—DHS spatial data repository —Shuttle Radar Topography Mission (srtm) rasters

All sources were included in the data lake and available to use. After data cleaning and pre-processing in our pilot, we only utilized the program data and census data. In later iterations, we incorporated all program, population, service delivery, and geographic data into a more comprehensive analysis but we do not discuss that in detail in this manuscript.

Our next task was to record and study the metadata and assess the quality of the records, both laborious and painstaking efforts that consumed most of our time and budget. For this task, we employed the ICPSR (ICPSR, 2020) metadata standards, based on the Dublin Core guidelines. This gave the team an opportunity to see at a glance the key information on the data in our lake and think about how to make them interoperable.

Across these data sets, we compiled several thousand variables. However, when we began to assess the quality of the variables we were faced with vast amounts of missing data. In the short amount of time at our disposal, we decided to eliminate all DHIS2 variables because we could not confidently impute missing values. Before machine learning models can be executed, data must be pre-processed to remove highly correlated variables (>0.80) and variables with near-zero variance that are not helpful in discriminating between categories. The iHRIS and Establishments data on staffing levels was also excluded due to low variation, e.g., few facilities meeting quotas. Remaining variables were transformed into dichotomous variables and then standardized. After this process of cleaning and pre-processing, we were left with 472 variables of the 1,200 we started with to feed into our analytical models.

### Analysis

We anticipated that most of our time would be spent collecting, cleaning, and wrangling data to merge it together before we would even be able to run a simple machine learning model. We therefore made an analytical memo describing our process and results a key deliverable of the project. While part of the team was focused on collecting and cleaning data, two team members with the most technical expertise in machine learning (AF and CE) collaborated to produce the analytical memo. The memo described literature on how machine learning methods had been applied to address problems like ours and various machine learning analytical approaches and their drawbacks including class imbalance on the outcome variables, missing data, and overfitting, and suggested analyses for when the data were cleaned and in hand for the project. This memo served as a way to communicate the intuition of machine learning and its limitations to the other members of the team who were new to these approaches and to make our efforts as transparent as possible. We added a description of the results of our analysis to the last section of the analytical memo.

In order to make our data interoperable, we merged all data at the facility level. Each facility was connected to its closest DHS sampling cluster using GPS coordinates and to the nearest parish in the Census using parish boundaries obtained by the project. This gave us the ability to use both facility-level data that USAID RHITES-E had collected and rich contextual information from population-based surveys.

Our primary initial analytical approach was hypothesis-free. This approach leveraged an unsupervised machine learning algorithm called k-means from the stats package in R (R Core Team, 2016). K-means does not require an outcome variable. Instead, it iterates over the data to identify clusters that minimize the difference within each cluster and maximize the difference between clusters. This approach is known as phenotyping (Basile and Ritchie, 2018) and provides groups of similar observations based on their features (e.g., hair and eye color). In this case, we were creating phenotypes of health facilities based on their characteristics like staffing, supplies, and services delivered. Once we had the results of this initial clustering, we could use cluster membership as an outcome variable. We then used a supervised machine learning process called recursive feature elimination (RFE) with a support vector machine (SVM) using a radial kernel from the caret package in R (Kuhn, 2019) to find the minimum number of variables that would correctly identify cluster membership.

Our initial clustering analysis returned four clusters as a reasonable mathematical solution. These four clusters could be accurately predicted using 50 of the variables we collected of the total 1,200 possible before pre-processing. The ability of machine learning to reduce the variables needed to review was a key success of this approach. With these results, we could quickly be pointed in fruitful directions for further analysis.

We shared the results of this analysis with our Uganda-based colleagues who were immediately able to understand the intuition behind the processes and start to interpret the results. Based on this initial analysis, the Uganda project leadership requested additional effort for our team in the next USAID RHITES-E work plan in order to investigate questions important to project implementation using our data lake. To date, two additional sprints have been approved and incorporated into the USAID RHITES-E activity budget.

## Discussion

Based on our successful experience of gaining buy-in, setting up an interdisciplinary team, building a data lake, and performing machine learning analysis, we are able to offer the following lessons learned to other practioners who are seeking to enhance their own work with machine learning but are unsure where to start.

### Set up the project to show value (or fail) quickly with minimal investment

Machine learning is a new approach that can be risky to take on among other available wellknown approaches. We found that by setting up the activity as a sprint of about 10 weeks, we were able to rapidly test our approach and show value leading to greater confidence in this approach from project leadership and additional requests for analysis. Key to our success was internal support from the IntraHealth's digital health team, where this project originated, to understand the value that can be unlocked by machine learning approaches and to endorse the initial pilot exercise.

### Find a data scientist who can lead the effort's technical approach

We built this initial approach by contracting a doctoral-level public health researcher from a local university with training in international population health and data science as a lead technical expert. We later hired her as a senior data scientist and placed her on the digital health team and since have grown the team to add an associate data scientist who had been working in the private sector but was attracted to international development by the lure of data and opportunities for improving health outcomes. While much of the work of machine learning can be carried out by a consultant, we are committed to increasing the use of data science at our organization, which requires a full-time employee to develop and iterate on these approaches.

### Build an interdisciplinary team and be prepared to communicate the value of machine learning with skeptics

Machine learning can be an intuitive process and data scientists should be able to explain the approach to non-technical audiences to build trust in the results of these methods. As data science continues to permeate the world of global health and development, a gap may emerge between the old guard with field expertise and newcomers trained in newly developed data science approaches. There will be many opportunities for collaboration to make machine learning approaches complementary to more traditional approaches by grounding these approaches in the local contexts in which we work.

### Minimize costs by using publicly available data; the main cost driver will be effort to clean and analyze data

Machine learning using population data is an extremely cost-effective approach to learn more about the contexts in which we support local ministries to reach their health targets. Nearly all of the data used in this analysis was available without cost for the project's use. The only exception to this was data from the project's baseline survey, which had already been financed by the project prior to the beginning of our activity. In addition, the DHS and census are widely available surveys that can be downloaded by researchers without fees. Ultimately, the ongoing cost to take on these projects is time for effort to complete the analysis.

## Conclusions

Our journey without a destination into building a data lake and conducting machine learning was merited by what the team developed and discovered. First, and most important, we could do it. We could bring together a critical mass of talent, data, and technology resources to conduct an investigation using machine learning tools and practices. We also learned that an *ad hoc* team could conduct a highly technical process with large amounts of data, many variables, and complex analyses in the virtual space. Finally, we proved the concept to project leadership in Uganda and the Activity invested in another sprint to investigate questions they provided that could directly enhance service delivery. To date, a third sprint has been approved by the project leadership in Uganda.

Machine learning is more accessible than is commonly perceived. The various technologies—computational, connectivity, storage, and accessibility—offer an onramp for other such journeys. The human resources reside in a cross-section of project management, computer science, statistics, information and communications technology, health informatics, measurement and evaluation, and data science. Working collaboratively with such a team brings each to contribute from their specific sector for the purpose in common. These are resources generally accessible in the domain of global digital health development.

## Data availability statement

The data analyzed in this study is subject to the following licenses/restrictions: This project used a combination of publicly available and proprietary data. Sources such as the Demographic and Health Surveys (DHS) and census are publicly available. Other sources such as IntraHealth International's baseline facility survey and Uganda Ministry of Health district level data on health facility performance are not publicly available.

## Author contributions

DP initiated the machine learning pilot on which this article is based. AF, IW, NMa, and CE cleaned the data and analyzed the results. RS and NMi provided informatics support. AF outlined and DP and AF wrote the initial draft of the manuscript. CK and WV provided oversight of the entire process. All authors have reviewed and approved the final version of the manuscript.

## Funding

Funding for this work was provided by IntraHealth International. The initial pilot did not support the project directly, but allowed us to strategically explore machine learning as a data service we could incorporate into our program delivery.

## Conflict of interest

The authors declare that the research was conducted in the absence of any commercial or financial relationships that could be construed as a potential conflict of interest.

## Publisher's note

All claims expressed in this article are solely those of the authors and do not necessarily represent those of their affiliated organizations, or those of the publisher, the editors and the reviewers. Any product that may be evaluated in this article, or claim that may be made by its manufacturer, is not guaranteed or endorsed by the publisher.
